# AN ACTION PLAN FOR ORGANIZATIONS SERVING OLDER ADULTS AND THEIR CAREGIVERS DURING PUBLIC HEALTH EMERGENCIES

**DOI:** 10.1093/geroni/igac059.1257

**Published:** 2022-12-20

**Authors:** Alycia Bayne, Lauren Isaacs, Jessica Fox, Rachel Singer, Roy Ahn, Desirae Leaphart, Felicia Cerbone, Madeleine Liotta

**Affiliations:** NORC at the University of Chicago, Bethesda, Maryland, United States; NORC at the University of Chicago, Chicago, Illinois, United States; NORC at the University of Chicago, Bethesda, Maryland, United States; NORC at the University of Chicago, Bethesda, Maryland, United States; NORC, Chicago, Illinois, United States; NORC at the University of Chicago, Chicago, Illinois, United States; NORC, Bethesda, Maryland, United States; NORC at the University of Chicago, Bethesda, Maryland, United States

## Abstract

During public health emergencies, it is critical to maintain the continuity of services and resources essential to health and safety. Public health emergencies can disproportionately affect older adults and their caregivers. Organizations, including community-based, faith-based, rural, and tribal organizations, can play a vital role during a public health emergency response given their familiarity with the community’s unique needs and resources. With support from the CDC Foundation and technical assistance from the Centers for Disease Control and Prevention, NORC at the University of Chicago conducted a study to identify public health interventions to meet the needs of older adults and their caregivers during public health emergencies. Methods included an extensive search of peer-reviewed and grey literature in Spanish and English to identify interventions on six topics: deconditioning; deferral of medical care; elder abuse and neglect; management of chronic conditions; social isolation; and caregiving. NORC identified 300 public health interventions to support older adults and their caregivers during public health emergencies with a focus on underserved populations, including programs to support racial and ethnic minority populations, people with disabilities, and rural and tribal communities. NORC developed Search. Find. Help., a virtual resource library, and an Action Plan to support organizations in using these interventions. Search. Find. Help., which houses the Action Plan, has had 34,000 unique users. This session focuses on how organizations that serve older adults and caregivers can use the Action Plan’s four phases to select, adapt, implement, and evaluate public health interventions before or during a public health emergency.

